# A Novel Injection Technique to Prevent Exacerbation of Sunken Cheek After Botulinum Toxin Type A Treatment for Masseter Hypertrophy: A Prospective Clinical Study

**DOI:** 10.1111/jocd.70120

**Published:** 2025-03-26

**Authors:** Shi‐Liu Huang, Yan Guo, Jing Liu, Dan Ye, Zhao‐Yang Wang, Hong‐Yan Gao, Yu‐Lin Dong

**Affiliations:** ^1^ Department of Dermatology and Aesthetic Medicine Xi'an Honghui Hospital Xi'an China; ^2^ Department of Medical Cosmetology Xi'an Jiaotong University College of Stomatology Xi'an China; ^3^ Department of Dermatology The Second Affiliated Hospital of Xi'an Jiaotong University Xi'an China; ^4^ Department of Dermatology Xi'an People's Hospital Xi'an China; ^5^ Department of Medicine Xi'an Jiaotong University College of Stomatology Xi'an China

**Keywords:** botulinum toxin type A, injection technique, masseter hypertrophy, sunken cheek

## Abstract

**Background and Objectives:**

Sunken cheek is often an undesirable aesthetic outcome, making the face appear gaunt and tired. It is also a common adverse effect of injections for treating masseter hypertrophy. This study aims to assess the efficacy of a novel injection technique in preventing sunken cheek after botulinum toxin type A (BoNT‐A) treatment for masseter hypertrophy, focusing on minimizing adverse aesthetic effects while ensuring effective masseter muscle reduction.

**Materials and Methods:**

The degree of sunken cheek was compared on both sides before injection and 4 weeks postinjection using the validated assessment scales for the midface by Jean Carruthers, etc. Telephone follow‐ups were conducted in weeks 1, 2, and 4 to assess subjective perceptions of sunken cheek, monitor adverse effects, and evaluate participant satisfaction with the procedure and the hospital.

**Results:**

Before treatment, there were nine participants with Grade 0, 62 with Grade 1, and 11 with Grade 2 sunken cheek. At week 4, the numbers were 13 with Grade 0, 59 with Grade 1, and 10 with Grade 2. No patients reported an aggravation of sunken cheek during follow‐up calls. Adverse reactions primarily occurred within the first 2 weeks and improved spontaneously.

**Conclusions:**

The novel BoNT‐A injection technique for masseter hypertrophy is safe and prevents the exacerbation of sunken cheeks. The results suggest that this method does not aggravate sunken cheeks and may even improve cheek depression severity in some individuals.

## 1 Introduction

Masseteric hypertrophy is a common benign condition characterized by the enlargement of the masseter muscles in the anterior portion of the mandibular bone [1]. This condition not only affects facial aesthetics but can also have a psychological impact on the patient [2]. Various factors can contribute to masseteric hypertrophy, including bruxism, hyperfunction of the masseter muscle, microtrauma, and daily habits [1, 3]. Masseteric hypertrophy adds width to the jawline, affecting the harmony of the facial contour, which is often considered unappealing, particularly in Asian aesthetics favoring delicate contours. In recent years, botulinum toxin type A (BoNT‐A) injections into the masseter muscle have become a common treatment option [3, 4, 5, 6].

BoNT‐A reduces muscle activity by blocking the release of acetylcholine, eventually resulting in a decrease in muscle volume [7, 8, 9, 10]. The application of BoNT‐A to the masseter muscle is highly effective, with noticeable volume reduction of the masseter within a few weeks after the injection [11, 12, 13]. However, it can also lead to complications such as chewing weakness, paradoxical bulging, sunken cheeks, and a sunken temporal fossa [14, 15].

Sunken cheek refers to the reduction in volume around the cheek area. In Caucasians, sunken cheek is often associated with physical health or slimness, whereas Asians typically interpret them as signs of fatigue or malnutrition. The incidence rate of sunken cheeks following BoNT‐A injections varies across different studies, ranging from 0.44% to 26.5%, and it usually resolves spontaneously within 1–8 weeks after the injection [14]. Studies have shown that sunken cheek is more common in patients with higher zygomas and less prominent cheek fat pads [16]. In addition, when the injection site is higher and the injection dose is greater, the occurrence of sunken cheek is higher [17].

Since 1994, researchers have been continuously optimizing injection techniques to minimize adverse reactions and complications in the treatment of masseteric hypertrophy with BoNT‐A injections. Despite significant improvements in injection techniques, sunken cheek remains a challenging complication to avoid entirely. To prevent this complication after BoNT‐A injection, we propose a new injection technique. This method, grounded in anatomical principles, redefines the injection range and injection site to optimize the use of BoNT‐A for the treatment of masseteric hypertrophy.

Our approach aims to provide valuable treatment experience and data for the future clinical application of BoNT‐A. In the future, as research continues to progress and techniques continue to improve, the therapeutic efficacy of BoNT‐A is expected to be further enhanced, while the incidence of complications will be significantly reduced. This will provide patients with safer and more effective treatment options, helping them achieve their desired facial aesthetic outcomes.

## 2 Materials and Methods

This was a prospective and evaluator‐blind clinical study. Written consent for photography release was secured prior to image capture. When necessary, patients were contacted via telephone to provide additional information or to authorize further photography. The study was conducted in compliance with the Declaration of Helsinki (1975).

### 2.1 Patients

We investigated a total of 45 patients treated with injections of BoNT‐A (botulinum toxin type A) with at least a 4‐week follow‐up from November 2023 to February 2024. The width of the lower third of faces is determined by the size and width of the mandible, as well as the thickness of the surrounding muscles and subcutaneous adipose tissue. Consequently, the selected patients are all with broad or square‐shaped lower faces due to masseter muscle hypertrophy instead of a wide mandible and full adipose tissue. Additionally, all patients had some degree of sunken cheeks before treatment.

The following patients will be excluded: (1) Patients with a history of facial fillers, facial lifting surgery, or other surgeries that could affect the assessment before treatment or during follow‐up; (2) Patients who have received BoNT‐A within the past year or have undergone therapy with intense pulsed light, laser, or radio frequency within the past 6 months; (3) Pregnant or breastfeeding women, or those planning to conceive within the next year; (4) Patients with depression or other mental illnesses; (5) Patients with liver or kidney dysfunction, or other visceral diseases.

### 2.2 Method of Treatment

#### 2.2.1 Injection Site and Landmarks

**FIGURE 1:**
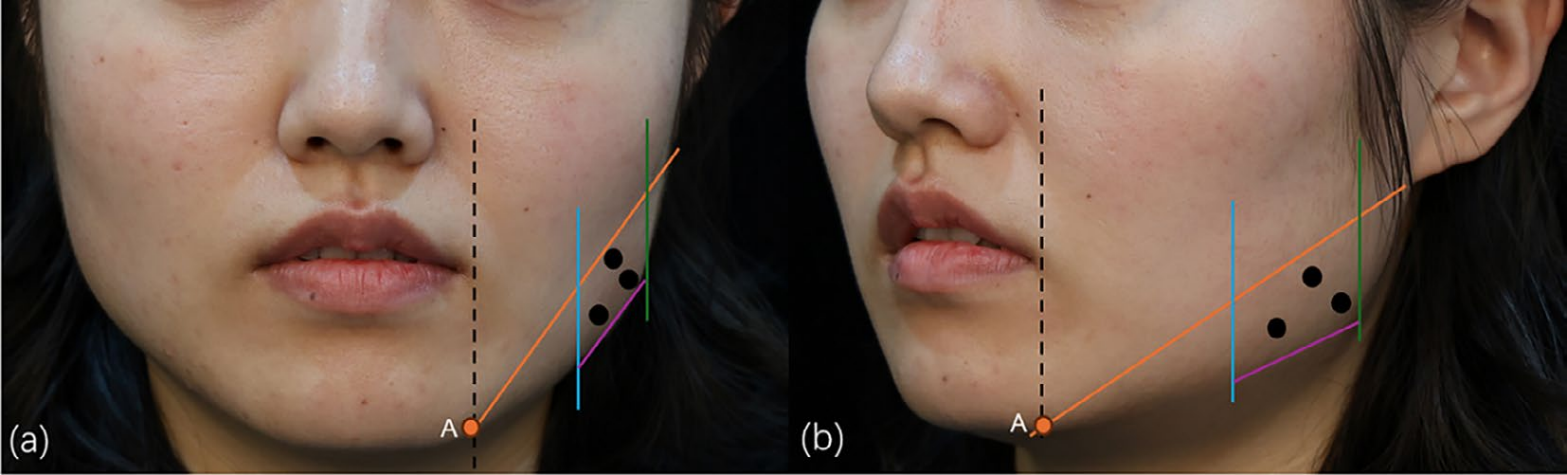
(a) Front and side injection diagrammatic sketch, (b) 45° side injection diagrammatic sketch. Black dashed line: the vertical line of the mouth corner; Point A: the junction of the black dashed line and the mandibular margin; Orange line: point A to the earlobe; Blue line: within 1 cm of the anterior margin of masseter muscle by palpation; Green line: within 1 cm of the posterior margin of masseter muscle by palpation; Purple line: mandibular margin; Point black: injection location.

Firstly, the main cause of a square face and the volume of the masseter muscle were estimated by physical examination. Secondly, we made an injection zone with four lines (Figure 1). The upper boundary of the zone is from the junction of the vertical line of the mouth corner and the mandibular margin to the earlobe; the lower boundary is within 1 cm of the border of the mandible, and the boundaries on both sides are within 1 cm of the anterior and posterior margins of the masseter muscle by palpation. Thirdly, we injected at three points, taking into consideration the diffusion of BoNT‐A. Fifty percent of the total dose was injected at the most prominent point, and each 25% of the total dose was injected separately at the other point 1 cm away from the most prominent point. It is noteworthy that the needle tip was inserted until it contacted the mandible and then withdrawn 1–2 mm to avoid injecting into the superficial facial muscles.

#### 2.2.2 Solution Preparation and Injection

BoNT‐A (Botox; Allergan Inc.) was reconstituted to a final concentration of 40 U/mL in sterile saline (100 U BoNT‐A + 2.5 mL sterile saline). Aliquots of 1 mL (equivalent to 40 U) were injected through a 1 mL insulin syringe (Shanghai Kangdelai Enterprise Development Group Co. Ltd., 0.33 mm × 13 mm RW LB). All injections are meticulously administered by an experienced plastic surgeon. Furthermore, the injection dose referred to Xie et al. [18] depended on the thickness, contractile force, and classification of the masseter muscle.

### 2.3 Evaluation Criteria

Patients were photographed by Canon (Canon EOS 5D Mark III) before treatment with BoNT‐A at Week 0 (baseline) and 4 weeks after treatment. The criterion for assessing the degree of sunken cheek is the validated assessment scales for the mid face by Jean Carruthers, etc., published in 2012 [19]. Whether the sunken cheek is aggravated depends on the comparison of digital pictures taken at Weeks 0 and 4 by two independent cosmetic dermatologists, as well as telephone follow‐ups at Weeks 1, 2, and 4. When the two dermatologists have divergent opinions, we will defer to the perspective of the third dermatologist. In addition, we also classified the masseter muscle based on the classification published in 2014 by Xie et al. [18].

The content of telephone follow‐up includes whether the patient feels that the sunken cheek has worsened after the injection, whether there are adverse reactions such as bruising, soreness, and swelling after the injection. At Week 4, we will ask the patient about their willingness to inject again in the future and their satisfaction with the treatment. The satisfaction of all participants was estimated based on a grading scale (0 = dissatisfied, 1 = somewhat satisfied, 2 = satisfied, 3 = very satisfied).

### 2.4 Statistical Analysis

Statistical analyzes were performed by SPSS software (version 19.0; SPSS Inc.). Wilcoxon signed‐rank test (Mann–Whitney *U*) was used in comparing the differences between treatment before and 4 weeks after treatment. *p* < 0.05 was considered statistically significant.

## 3 Results

### 3.1 Demographic and Baseline Characteristics

This study included a total of 45 patients, encompassing 90 hemifacial regions; all patients were female. The average patient age was 27.47 ± 4.34 years (range, 21–38 years) (Table 1). According to the validated mid‐face assessment scales by Jean Carruthers et al. [19], there were 11 hemifacial regions (12.22%) classified as grade 0, 67 regions (74.44%) as grade 1, and 12 regions (13.33%) as grade 2. The masseter muscle was categorized into five types (I–V), with 16 type I (17.77%), 58 type II (64.44%), 4 type III (4.44%), 0 type IV, and 12 type V (13.33%) in this study. The injection dosage was determined based on the patient's masseter muscle thickness, contracted strength, classification, and the degree of sunken cheek, as detailed in Table 1.

**TABLE 1:** Baseline demographic characteristics of patients plus injection dosage.

Characteristics	Value
Female, *n* (%)	45 (100.00)
Age (years), mean ± SD	27.47 ± 4.34
Classification of sunken cheek, *n* (%) & average injection dosage (U), mean ± SD	
0	11 (12.22) & 30.00 ± 2.86
1	67 (74.44) & 30.10 ± 3.75
2	12 (13.33) & 28.50 ± 2.71
3	0
4	0
Classification of the contracted masseter muscle, *n* (%) & average injection dosage (U), mean ± SD	
I	16 (17.77) & 28.25 ± 2.46
II	58 (64.44) & 29.53 ± 2.12
III	4 (4.44) & 31.75 ± 1.71
IV	0
V	12 (13.33) & 34.33 ± 2.53

Abbreviation: SD, standard deviation.

### 3.2 Sunken Cheek Comparison and Willingness to Inject Again

**FIGURE 2:**
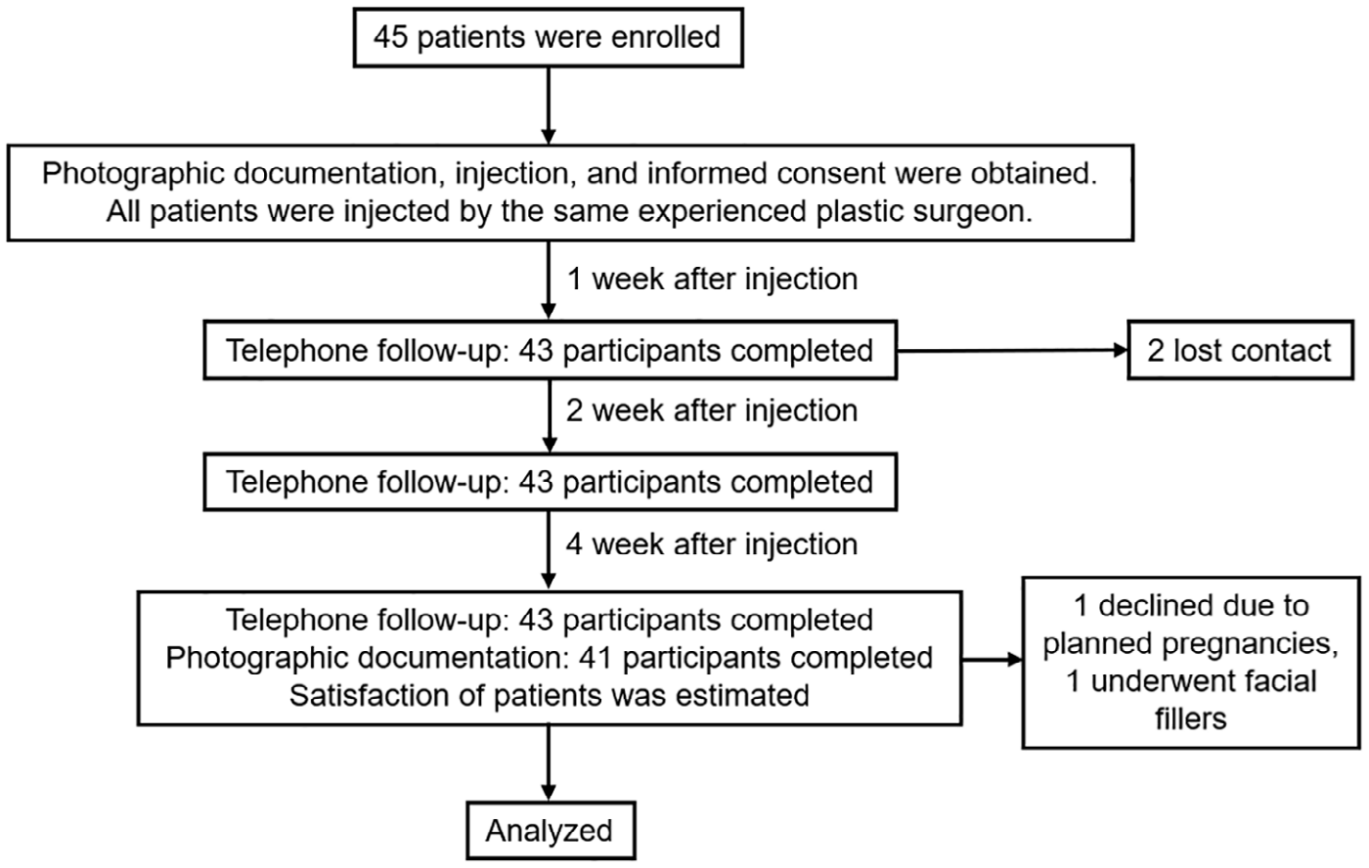
Clinical trial flowchart.

A total of 43 participants completed the telephone follow‐up, with two lost to follow‐up within a 1 week postprocedure. At the 4‐week follow‐up, 41 participants expressed a willingness to undergo a repeat injection. Among these, one participant declined due to planned pregnancies, and one underwent facial fillers, and they were not willing to take photos again. At Week 4, 41 participants completed the photographic follow‐up (Figure 2).

**FIGURE 3:**
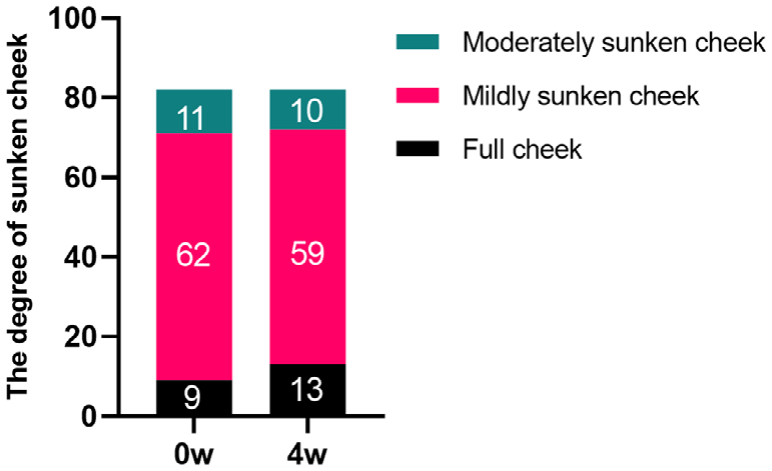
The degree of sunken cheek at Week 0 and Week 4. The degree of sunken cheek was estimated according to the validated midface assessment scales by Jean Carruthers et al.

**FIGURE 4:**
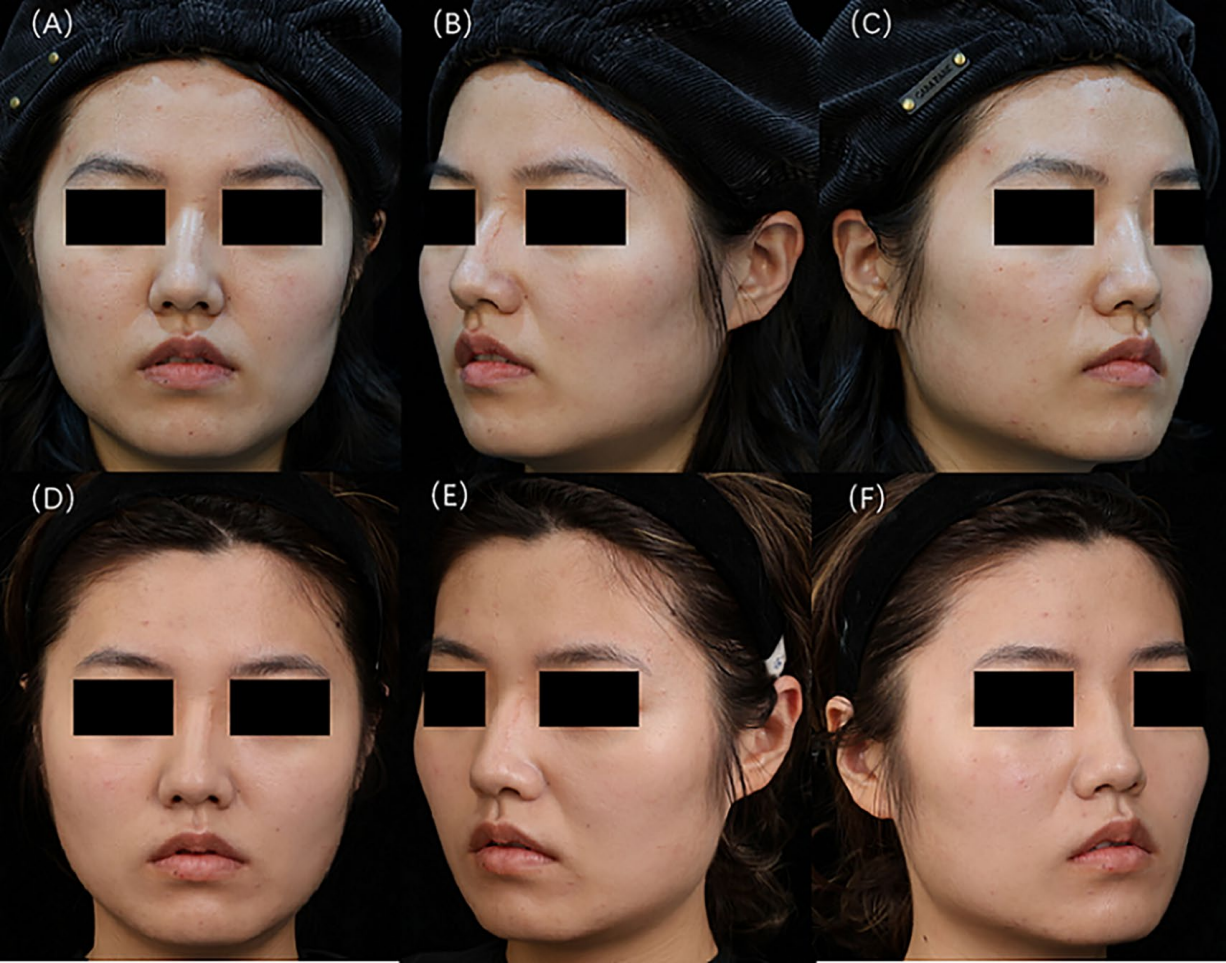
A 24‐year‐old girl. Injection dosage: left side 31 U, right side 33 U. (A) Frontal views before treatment; (B) 45° left profile before treatment; (C) 45° right profile before treatment; (D) frontal views at 4 weeks postinjection; (E) 45° left profile at 4 weeks postinjection; (F) 45° right profile at 4 weeks postinjection.

**FIGURE 5:**
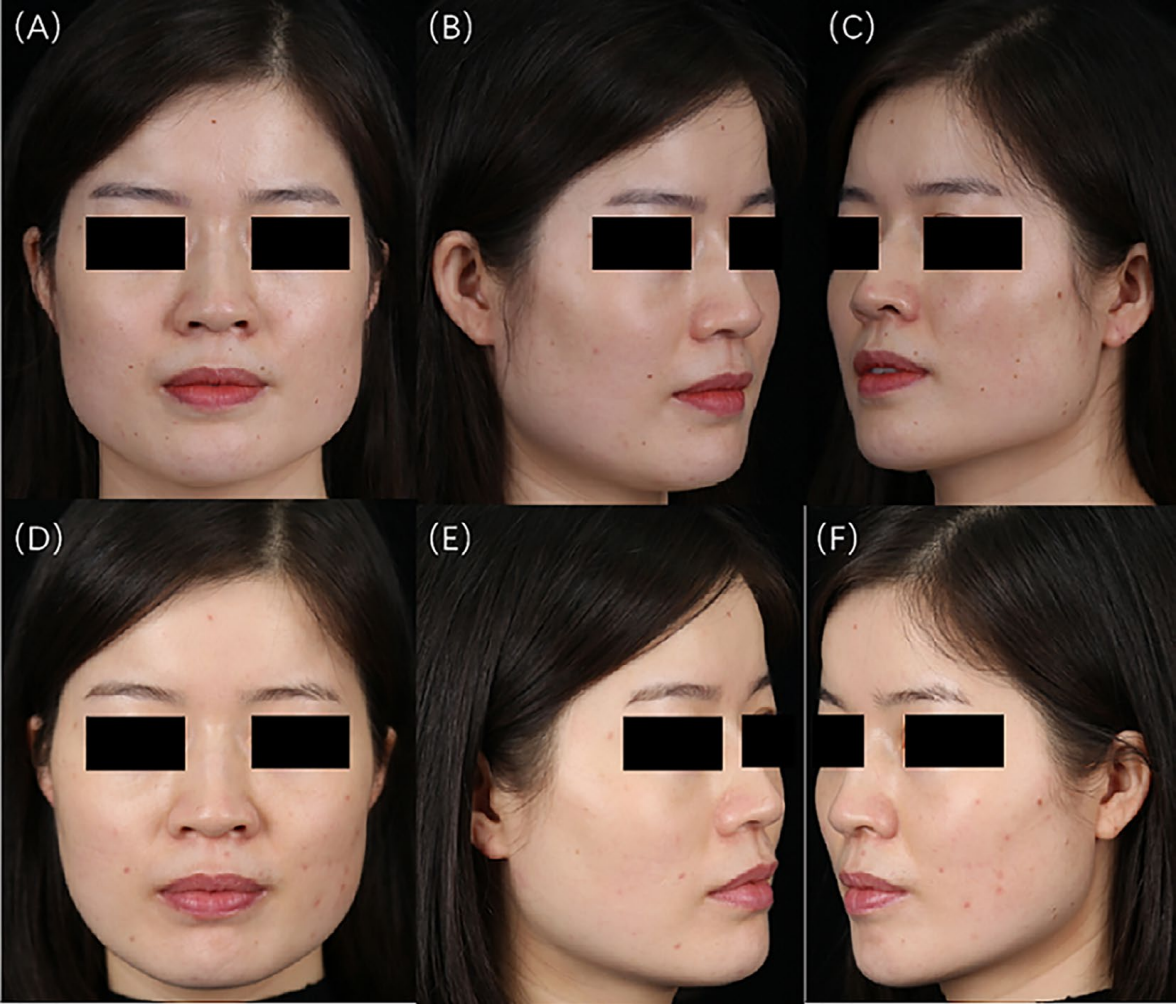
A 33‐year‐old girl. Injection dosage: left side 36 U, right side 36 U. (A) Frontal views before treatment; (B) 45° left profile before treatment; (C) 45° right profile before treatment; (D) frontal views at 4 weeks postinjection; (E) 45° left profile at 4 weeks postinjection; (F) 45° right profile at 4 weeks postinjection.

Prior to treatment, evaluations by three cosmetic dermatologists revealed 9 cases of grade 0 sunken cheek, 62 cases of grade 1, and 11 cases of grade 2. Four weeks postinjection, the distribution changed to 13 cases of grade 0, 59 cases of grade 1, and 10 cases of grade 2. Notably, 4 cases of mildly sunken cheek improved to full cheek, and 1 case of moderately sunken cheek improved to mildly sunken cheek. However, there were no statistically significant differences between the groups (*p* > 0.05) (Figure 3). The data suggest that an appropriate injection technique may not only prevent the exacerbation of sunken cheek but also lead to its improvement. Typical cases are illustrated in Figures 4 and 5.

### 3.3 Side Effects and Patient's Satisfaction

During the 8‐week follow‐ups, a total of 43 patients provided documentation of adverse events. The primary adverse events included bruising, swelling, masseter soreness and weakness, and paradoxical masseteric bulging, most of which occurred within the first 2 weeks postinjection. All these side effects resolved spontaneously without any intervention (Table 2).

**TABLE 2:** Side effects during the study.

	Number	Occurrence time	Duration (days)
Bruising	1	Immediately after injection	7
Swelling	1	1 day after injection	2
Soreness and weakness	3	3 days after injection	5
		1 day after injection	3
		1 day after injection	2
Paradoxical masseteric bulging	2	2 days after injection	5
		1 day after injection	7

**FIGURE 6:**
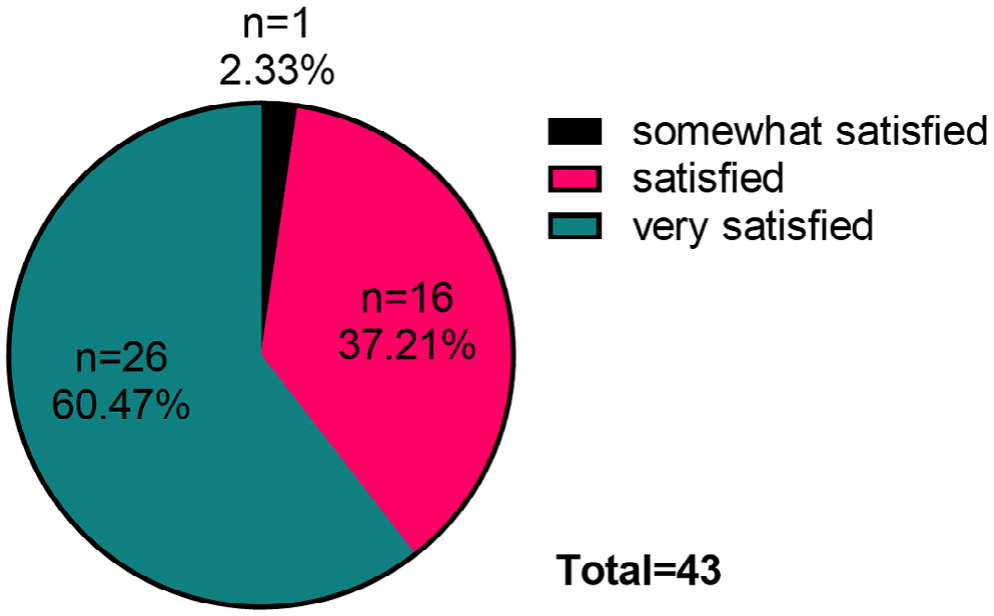
Percentage distribution pie chart: satisfaction of 43 patients.

At the 4‐week postinjection, one person was somewhat satisfied with the injection results, 16 were satisfied, and 26 were very satisfied (Figure 6).

## 4 Discussion

Sunken cheek is commonly regarded as an adverse effect of masseter injections [14]. Anatomically, it is primarily attributed to high cheekbones, insufficient prominence of the buccal fat pad, and loss of facial volume [12, 18, 20]. Additionally, technical issues such as excessive injection dosage and placement at higher sites can exacerbate sunken cheek [17]. In response, we developed a novel injection technique designed specifically for patients who are presenting with preexisting sunken cheek and seek to avoid its aggravation.

Currently, to minimize the impact on the surrounding facial muscles and glands near the masseter, the commonly designated safe injection zones in clinical practice are defined by the tragus–mouth line, the mandibular border, and a distance of 1 cm away from both the anterior and posterior margins of the masseter muscle, and the injection sites may range from a single point to as many as six based on the specific circumstances [5, 14, 16, 21, 22].

This technique's key innovation involves redefining the upper boundary of the masseter injection area, thereby improving masseter hypertrophy while mitigating the risk of worsening cheek depression.

The injection dosage reference in this study was primarily derived from the 2014 investigation by Xie et al. Through statistical analysis of 504 masseter muscles, their research established thickness‐based dosage recommendations: an average dose of 20–25 units for 10 mm thickness, 25–30 units for 10–13.9 mm thickness, and 30–40 units for thickness exceeding 14 mm [18].

Due to technical limitations in the current study, precise measurement of individual masseter thickness was not feasible. However, we successfully implemented an alternative approach through masseter bulging type classification. Xie et al.'s data demonstrated overlapping thickness ranges across bulging types: Type I (5.1–17.9 mm), Type II (7.1–18.7 mm), Type III (5.2–18.3 mm), Type IV (8.0–18.3 mm), and Type V (14.0–19.9 mm) [18]. Leveraging this classification system combined with clinical experience, a skilled plastic surgeon developed personalized injection protocols.

In this study, telephone 4‐week follow‐ups were completed for 43 patients, and photographic assessments were conducted for 41 patients. The study outcomes revealed the following dose distributions: Type I bulging (*n* = 16) received 28.25 ± 2.46 U, Type II (*n* = 58) 29.53 ± 2.12 U, Type III (*n* = 4) 31.75 ± 1.71 U and Type V (*n* = 12) 34.33 ± 2.53 U. Notably, no Type IV bulging cases were observed in our study. This methodology demonstrates that bulging type classification, when integrated with expert clinical judgment, serves as a viable alternative for determining appropriate botulinum toxin dosage in masseter hypertrophy treatment, particularly when direct thickness measurement is unavailable. The dose escalation pattern across bulging types (Type I to Type V) aligns with the positive correlation between anatomical protrusion and muscle volume observed in previous anatomical studies.

Furthermore, the analysis of photographic data revealed that within 4 weeks postinjection, one case of sunken cheek with masseter hypertrophy demonstrated improvement from moderate to mild severity, while four cases improved from mild to full. This new technique not only avoided exacerbating cheek depression but also reduced it in some patients. During the telephone follow‐ups at weeks 1, 2, and 4, none of the 43 patients reported any worsening of sunken cheek [17]. Additionally, 37.21% of patients expressed very satisfied with the injection outcomes at 4 weeks after injection, 60.47% expressed satisfied, and 2.33% expressed somewhat satisfied.

These results demonstrate that the refined injection method effectively mitigates the adverse effects of masseter injections on sunken cheeks. This finding is of significant clinical relevance, indicating that optimizing injection techniques can not only enhance masseter hypertrophy but also preserve the natural appearance of other facial areas, avoiding adverse effects such as sunken cheeks. This approach offers valuable insights for clinical practice in masseter injections and provides a foundation for future refinements in injection techniques to improve patient satisfaction.

While this study offers meaningful insights, several limitations should be noted. The short follow‐up period restricts a thorough evaluation of long‐term efficacy and potential delayed side effects, possibly affecting the accuracy of results. Additionally, the small sample size, exclusively female participants, may limit the statistical power and generalizability of the findings, hindering a comprehensive understanding of diverse populations. The absence of photos from weeks 1 and 2 postinjection also raises concerns about the reliability of subjective assessments. Future studies should address these issues by extending follow‐up periods, increasing sample sizes, and improving data collection methods.

## 5 Conclusion

This study presents a new masseter injection technique designed to minimize sunken cheeks in patients with existing conditions. The technique avoids worsening sunken cheeks and even improves them in some cases. Follow‐ups revealed high patient satisfaction with the results. These findings suggest that the refined method effectively balances masseter hypertrophy with aesthetic outcomes, offering valuable insights for clinical practice.

## Author Contributions

Yu‐Lin Dong and Shi‐Liu Huang: design. Yu‐Lin Dong: injector. Jing Liu, Dan Ye, and Zhao‐Yang Wang: raters. Shi‐Liu Huang, Yan Guo, Hong‐Yan Gao: data analysis/writing. Yu‐Lin Dong: manuscript revision. Yu‐Lin Dong: supervision. All authors approved this final version.

## Conflicts of Interest

The authors declare no conflicts of interest.

## Data Availability

The data that support the findings of this study are available on request from the corresponding author. The data are not publicly available due to privacy or ethical restrictions.
